# A Severe Case of Minocycline-induced Hyperpigmentation of the Lower Extremities

**DOI:** 10.7759/cureus.2672

**Published:** 2018-05-22

**Authors:** Eric D Schadler, Thomas L Cibull, Stephanie L Mehlis

**Affiliations:** 1 Pritzker School of Medicine, University of Chicago; 2 Department of Pathology and Laboratory Services, NorthShore University HealthSystem; 3 Department of Medicine, Division of Dermatology, NorthShore University HealthSystem

**Keywords:** adverse drug reactions, hyperpigmentation, minocycline induced hyperpigmentation

## Abstract

Drug reactions are a common cause of cutaneous eruptions. The authors present a case of shin hyperpigmentation resulting from long-term minocycline treatment. This case illustrates a severe example of minocycline-induced pigmentation and reminds clinicians who prescribe this commonly used antibiotic to remain vigilant of this rare adverse reaction.

## Introduction

Minocycline-induced hyperpigmentation (MIH) is a cosmetically disfiguring drug reaction. It is most often reported in patients diagnosed with dermatologic, rheumatic, and orthopedic conditions. Pigmentation is thought to be the result of insoluble minocycline-iron complexes, and it may affect numerous body sites including skin, thyroid, bones, fingernails, toenails, oral cavity, and eyes [1]. The incidence ranges from low in patients with acne vulgaris (2.4%), to high in patients with rheumatoid arthritis (36%) or with chronic infections receiving long-term therapy (54%) [2-4]. Studies have proposed that higher dosage and advanced age may be associated with increased risk of hyperpigmentation; however, current literature is limited to smaller case reports and has not fully supported these findings [2-3].

## Case presentation

An obese 70-year-old man presented with rapidly progressive, asymptomatic pigmentation of six-months' duration affecting his lower extremities. The pigmentation started in a small area on his calf and spread rapidly. His medical history was significant for uncontrolled type-two diabetes mellitus, hypertension, and chronic back pain. Following a back surgery, the patient experienced an infected hematoma with S. aureus and suffered from recurrent S. aureus infections. As a result he was managed with 100 mg prophylactic minocycline twice daily for six years.

**Figure 1:**
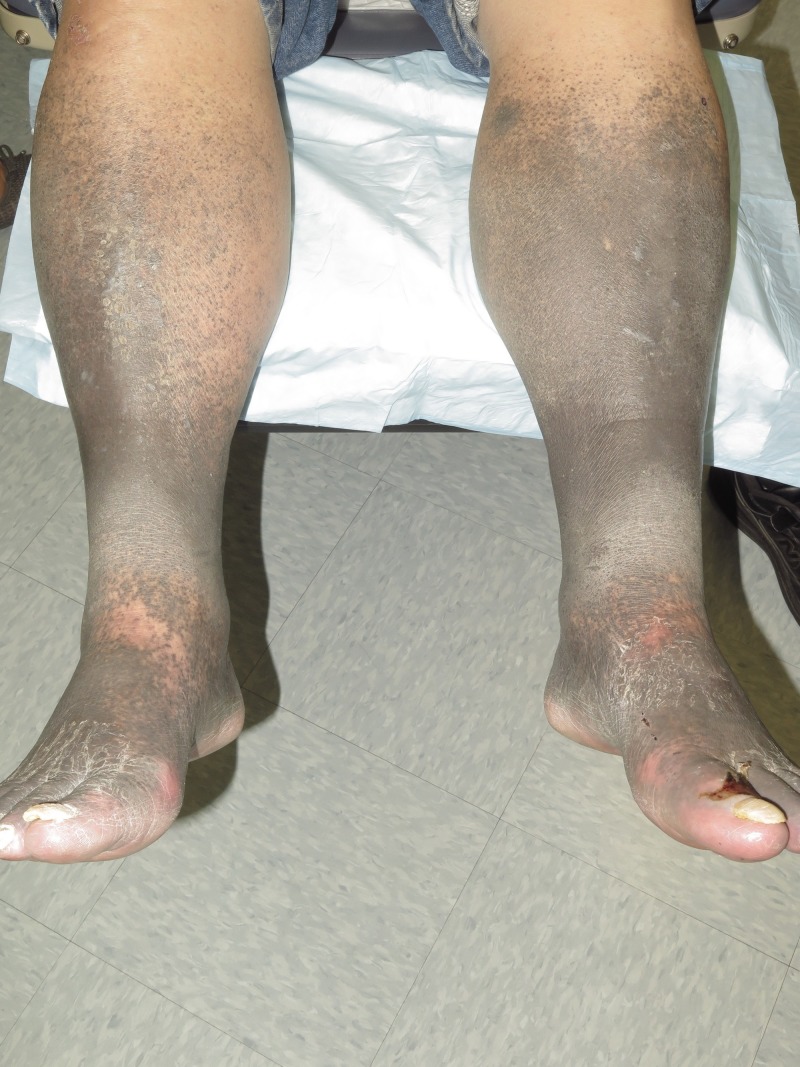
Lower extremity hyperpigmentation Diffuse blue-grey pigmentation extended along bilateral shins. The perifollicular distribution can be seen at the proximal extensor aspect of his legs.

**Figure 2:**
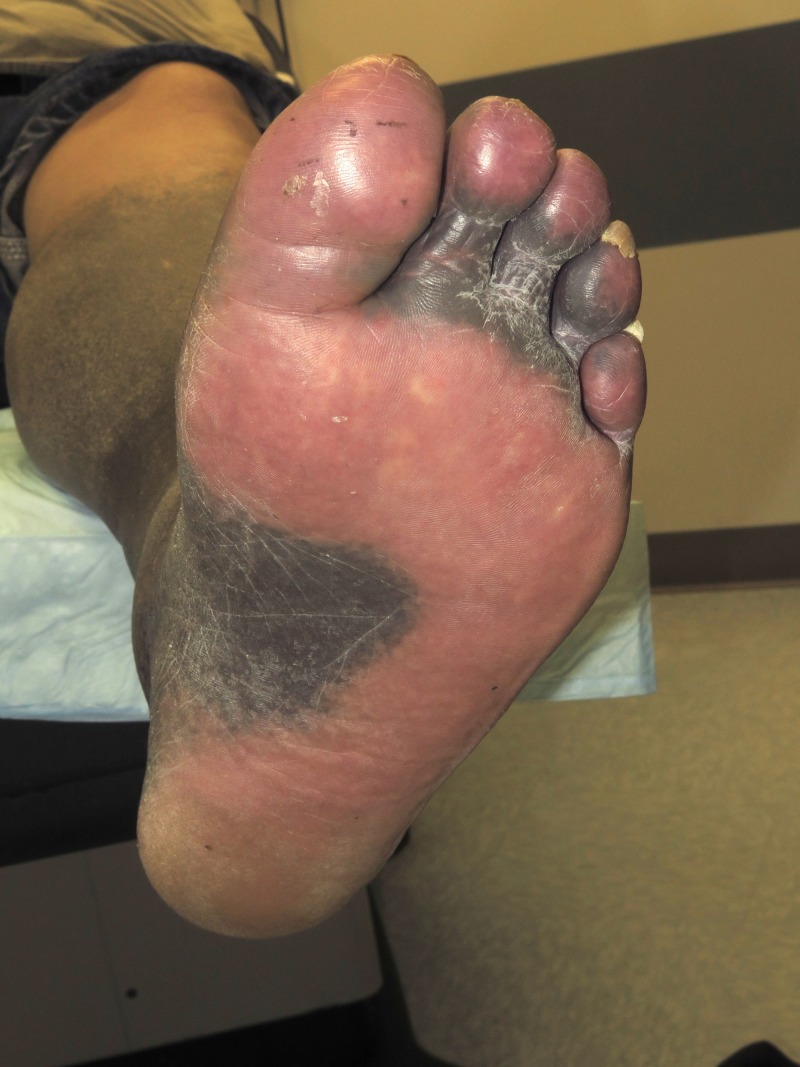
Pigment distribution on the sole Weight-bearing surfaces of soles and digits were spared from pigmentation changes.

On physical examination, blue-grey hyperpigmentation extended below the knees into a confluent patch over bilateral shins and dorsal surfaces of the feet (Figure 1). Evaluation of the soles revealed heavy pigmentation in the non-dependent areas with complete sparing of weightbearing surfaces (Figure 2). A perifollicular pigment distribution was noted near the proximal extensor aspect of his legs. No other areas of skin were involved including palms, body creases, or oral mucosa.

**Figure 3:**
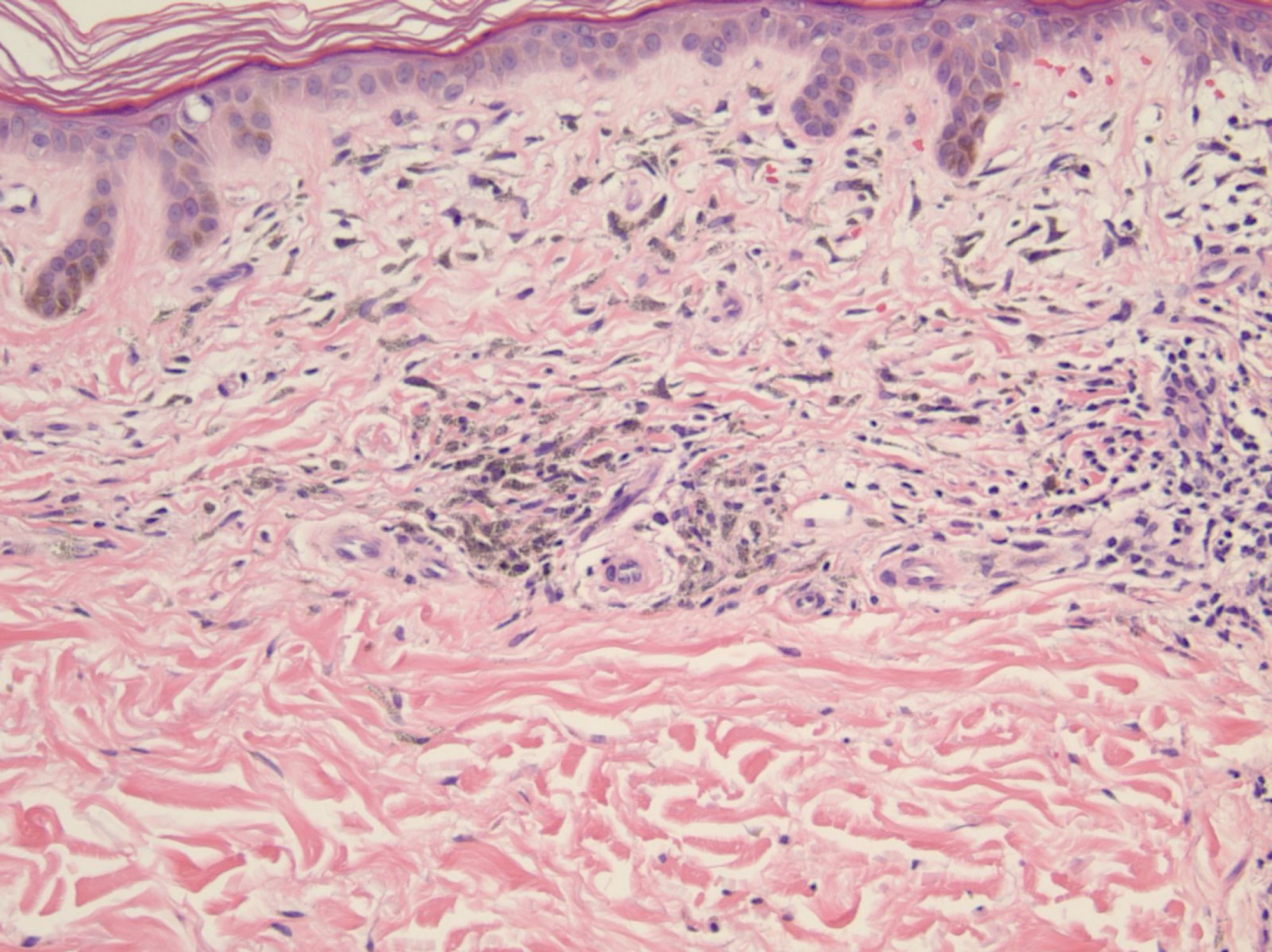
H&E stain at 20x magnification Histology revealed pigment containing, perivascular macrophages within the papillary and superficial reticular dermis.

**Figure 4:**
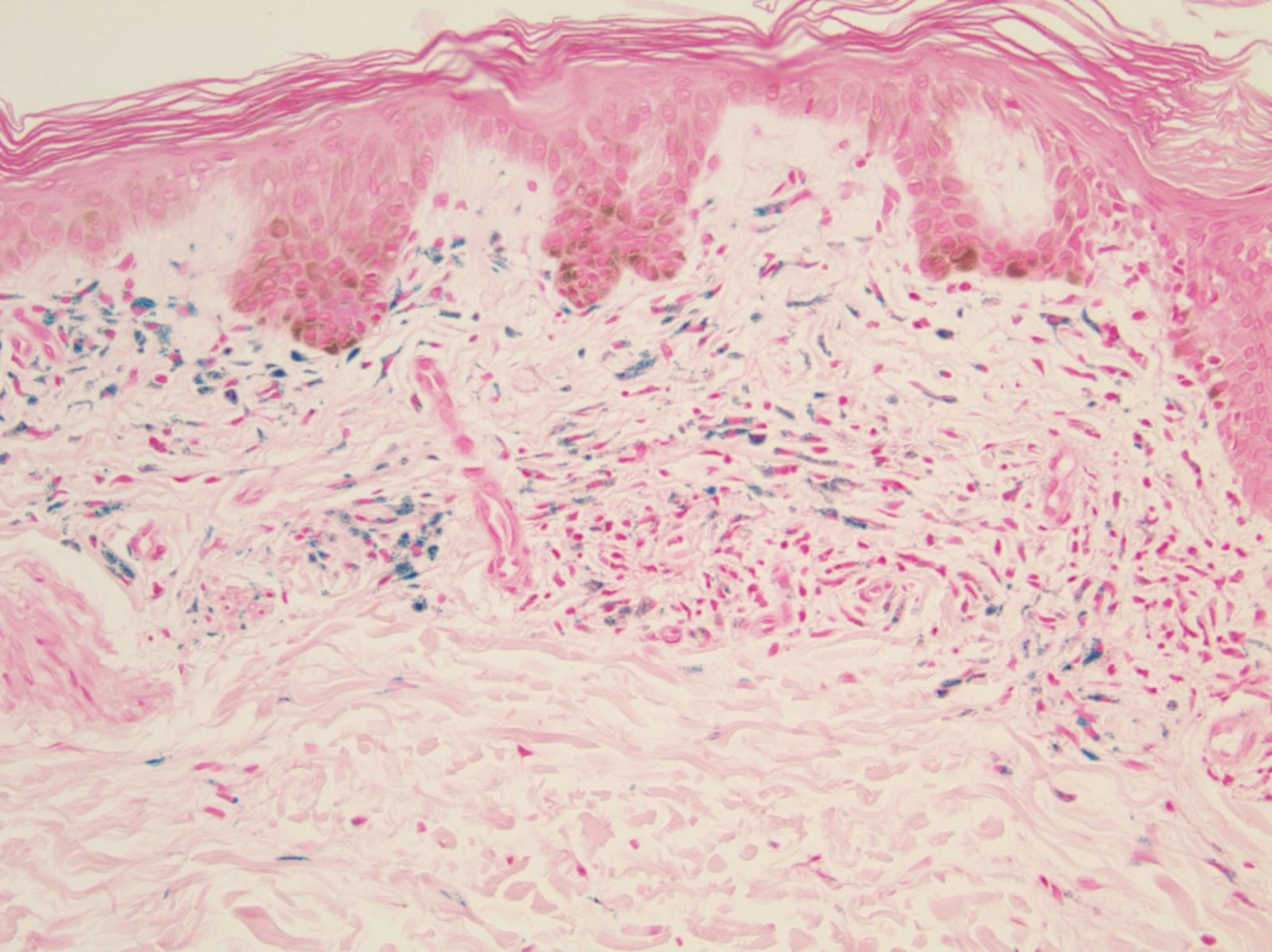
Perl's stain at 20x magnification Pigment stained positive using the Perl's Prussian blue method of staining.

**Figure 5:**
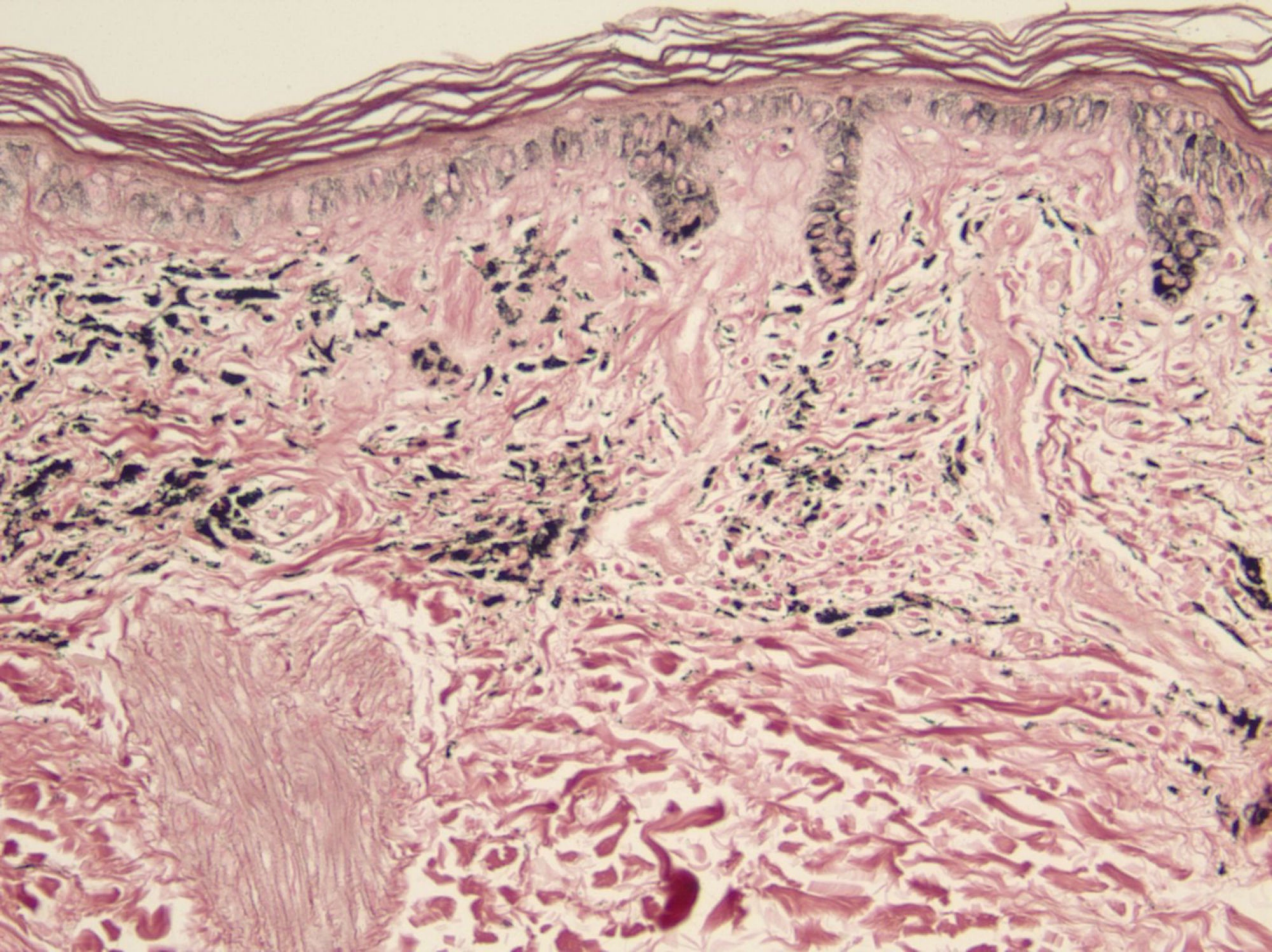
Fontana-Masson stain at 20x magnification The complex pigment stained positive with Fontana-Masson method of staining.

On histopathology, the papillary and superficial reticular dermis contained pigment in perivascular macrophages and within dermal dendrocytes (Figure 3). The complex pigment was positive with both the Perl's Prussian blue method for iron (Figure 4) and the Fontana-Masson method for melanin (Figure 5).

After discussion of the available treatment options, the patient opted not to pursue therapy given the intensity of pigmentation and his significant comorbidities.

## Discussion

Traditionally, three types of MIH have been recognized with a fourth being described in two case reports [5]. Type 1 occurs on the face in areas of scarring and prior inflammation. The blue-black pigment stains positive with Perl's Prussian blue staining (iron) and can be found within the dermis, scar tissue, and intracellularly in macrophages. Type 2 is blue-grey pigmentation occurring in normal skin frequently on the lower extremities. Perl's Prussian blue and Fontana-Masson (melanin) stains are positive in this type. The pigment is located in the dermis, surrounding fat, and within macrophages and myoepithelial cells. Type 3 is diffuse and involves normal skin. It is accentuated by sun-exposure and muddy brown in color. The pigment stains positive for melanin, but not iron, and is located in the epidermis, dermis, and within macrophages. Lastly, type 4, involves blue-grey pigmentation occurring in areas of scarring on the back. The pigment is positive with von Kossa staining for calcium in addition to melanin staining; it is located in the dermis, scar tissue, within macrophages and fibroblasts. In this case, the sparing of pigmentation on weight-bearing surfaces of the soles and digits (Figure 2) is a unique component and previously unreported. It is unclear if this pattern is of significance or an extraneous finding.

The differential diagnosis of hyperpigmentation disorders includes systemic diseases such as Addison’s disease and hemochromatosis, medications such as minocycline and amiodarone, and primary skin diseases such as pigmented purpuric dermatosis. A thorough physical examination to document all affected areas may limit the differential based on the distribution of pigmentation. Histologic examination can be helpful to identify the location of pigment, patterns of staining, and features that would be suggestive of a particular condition.

Addison’s disease, or primary adrenal insufficiency, is a state of hypocortisolism. Hyperpigmentation is diffuse and characteristically affects surfaces such as the palmar creases and oral mucosa. The pathogenesis of this pigmentation is due to the lack of negative feedback on the hypothalamus by cortisol resulting in increased cleavage of pro-opiomelanocortin into adrenocorticotrophic hormone (ACTH) and melanocyte-stimulating hormone (MSH). MSH stimulates melanocytes to increase melanin production. Addison’s disease is easily differentiated from MIH by the associated systemic symptoms that may include fatigue, weakness, weight loss, nausea, vomiting, and abdominal pain. In addition, patients may be hypotensive, hypoglycemic, and crave salt. Laboratory examination to measure serum ACTH and cortisol can help with diagnosis.

Hemochromatosis is a genetic condition resulting in iron overload. In one study of 100 patients with the disease, 98% displayed signs of abnormal skin pigmentation [6]. Classically, skin is described as having a bronze hue and pigment is generalized. The difference in color and location of pigmentation should be an early clue to differentiate hemochromatosis from MIH. The mechanism of pigmentation is due to deposition of hemosiderin, an iron storage complex, in the skin. Features of hemochromatosis include diabetes, liver disease, skin pigmentation, and cardiac disease. Measurement of serum ferritin and transferrin saturation is the first step in diagnosis followed by genetic testing for the HFE gene responsible for the disease.

Approximately 10 to 20% of cases of hyperpigmentation can be attributed to a drug [7]. Pathogenesis depends on the drug, but it may be explained by increased melanin production, synthesis of pigments such as lipofuscin, deposition of iron, or accumulation of drug complexes. Clinical features typically involve a slow, progressive pigmentation occurring over months or years. Common culprits include non-steroidal anti-inflammatory agents, antimalarials, antipsychotics, amiodarone, tetracyclines, silver, and gold [7]. A thorough medication history to explore new and chronic drug exposures is critical in diagnosing a drug-induced hyperpigmentation. Adverse drug reactions can be difficult to diagnose and such diagnosis relies on careful evaluation of the causal relationship between a drug and event. Naranjo et al. attempted to create a simple and reliable method for assessing the likelihood of an adverse drug reaction [8]. Their assessment, known as the Naranjo score accounts for multiple factors to estimate the probability of a drug reaction as “doubtful”, “possible”, “probable”, or “definite” (Table 1). Using the Naranjo score in this case estimated the likelihood of a drug reaction as “probable”. Limitations with this scoring system exist. In our case, questions 3, 4, 6, 7, 8, and 9 were either not able to be answered or not applicable to the situation. For example, MIH rarely will self-resolve upon discontinuation, especially with the severity seen in our case.

**Table 1:** Naranjo score for determination of an adverse drug reaction Total score less than 2: “doubtful”, 2 to 4: “possible”, 5 to 8: “probable”, and 9 or greater: “definite”.

	Yes	No	Do not know
Are there previous conclusive reports on this reaction?	+1	0	0
Did the adverse event appear after the suspected drug was administered?	+2	-1	0
Did the adverse drug reaction improve when the drug was discontinued or an antagonist was administered?	+1	0	0
Did the adverse drug reaction reappear when the drug was readministered?	+2	-1	0
Are there alternative causes (other than the drug) that could on their own have caused the reaction?	-1	+2	0
Did the reaction reappear when a placebo was given?	-1	+1	0
Was the drug detected in the blood (or other fluids) in concentrations known to be toxic?	+1	0	0
Was the reaction more severe when the dose was increased, or less severe when the dose was decreased?	+1	0	0
Did the patient have a similar reaction to the same or similar drugs in any previous exposure?	+1	0	0
Was the adverse event confirmed by any objective evidence?	+1	0	0

Lastly, a clinician should consider a pigmented dermatosis when evaluating a patient with hyperpigmentation. Pigmented purpuric dermatosis can be divided into five types and requires biopsy for differentiation. Clinically, these conditions are characterized by petechiae and or purpura on the lower extremities [9]. These disorders may have a range of appearances making diagnosis more challenging and necessitating histologic examination. Biopsy would demonstrate hemosiderin by iron stain and would be negative by Fontana-Masson.

Treatment of minocycline-induced hyperpigmentation relies on early recognition and discontinuation of the medication. In many cases, pigmentation will not self-resolve despite discontinuation of the drug. Therapeutic options are limited and currently involve the use of laser treatments.

## Conclusions

Minocycline-induced hyperpigmentation is a difficult-to-treat drug reaction that may result in cosmetically disfiguring consequences. Given the frequency of prescribing minocycline, physicians should be cautious to observe for any pigmentation changes. This is especially true in patients prescribed high doses or long courses of the antibiotic.
